# miR-24-3p promotes colon cancer progression by targeting ING1

**DOI:** 10.1038/s41392-020-0206-y

**Published:** 2020-08-25

**Authors:** Zhiying Gao, Likun Zhou, Shiyu Hua, Huan Wu, Laizhi Luo, Libo Li, Shuping Wang, Yanqing Liu, Zhen Zhou, Xi Chen

**Affiliations:** 1 State Key Laboratory of Pharmaceutical Biotechnology, Collaborative Innovation Center of Chemistry for Life Sciences, Jiangsu Engineering Research Center for MicroRNA Biology and Biotechnology, NJU Advanced Institute for Life Sciences (NAILS), School of Life Sciences, Nanjing University, Nanjing, Jiangsu 210046 China; 2 Research Institute of Medicine and Pharmacy, Qiqihar Medical University, Qiqihar, Heilongjiang 161006 China; 3 The Department of Ultrasound of Sun Yat-Sen Memorial Hospital, Sun Yat-Sen University, Guangzhou, Guangdong, 510000 China; 4 The Sixth Clinical Medical College of Capital Medical University, Beijing, 100029 China; 5 The First Hospital of Qiqihar, Qiqihar Medical University, Qiqihar, Heilongjiang 161006 China

**Keywords:** Gastrointestinal cancer, Cancer

**Dear Editor,**

Activation or upregulation of oncogenes and/or loss of function or downregulation of tumour suppressors are central to the processes involved in the transformation from normal colonic mucosa to malignant tumours. Inhibitor of growth 1 (ING1) has been functionally linked to cell cycle arrest, apoptosis and chromatin remodelling.^1^ Deregulated ING1 expression is a crucial event in haematological malignancies and solid tumours, such as breast,^2^ lung^3^ and colorectal cancer.^4^ Despite advances in identifying ING1-controlled cellular functions, ING1 regulation during colon cancer development remains largely unknown.

We first detected ING1 expression in 16 pairs of human colon cancer tissues and normal adjacent cancer tissues. The data showed that ING1 protein levels were dramatically decreased in colon cancer tissues compared with normal adjacent cancer tissues (Fig. 1a, Supplementary Fig. S1a), but ING1 mRNA levels were not significantly different between the two tissue types (Supplementary Fig. S1b). Pearson correlation scatter plot analysis showed no significant correlation between the ING1 protein and mRNA levels (Supplementary Fig. S1c). These results indicated that ING1 functions as a tumour suppressor in colon cancer.

**Fig. 1:**
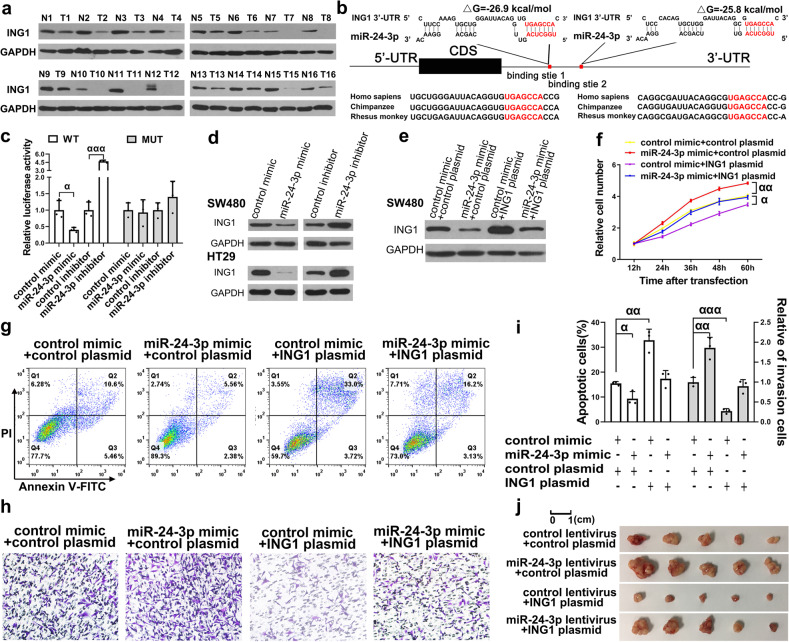
miR-24-3p promotes colon cancer proliferation and invasion and inhibits colon cancer apoptosis by targeting ING1. **a** Western blot analysis of ING1 protein levels in 16 paired colon cancer (T) and normal adjacent tissue (N) samples. **b** Potential binding site of miR-24-3p at the ING1 3′-UTR. **c** The relative luciferase activities in SW480 transfected with miR-24-3p mimic or inhibitor. **d** ING1 was negatively regulated by miR-24-3p in SW480 and HT29 cells. **e** The ING1 plasmid could effectively restored the ING1 protein level suppressed by miR-24-3p. **f–i** miR-24-3p promotes SW480 proliferation and invasion and inhibits SW480 apoptosis in vitro by targeting ING1 (**f**) CCK8 assay; (**g**) Apoptosis assay; (**h**) Transwell invasion assay; (**i**) Quantitative analysis of apoptosis and transwell invasion assays. **j** miR-24-3p promotes colon cancer growth in vivo by targeting ING1. Data are showen as mean ± s.e.m. in (**c**, **f** and **i**) with data points from independent experiment in (**c** and **i**). α *p* < 0.05; αα *p* < 0.01; ααα *p* < 0.001

To investigate the potential role of ING1 in the development of colon cancer, we transfected SW480 cells with siRNAs to silence ING1 expression and an overexpression plasmid to increase ING1 expression (Supplementary Fig. S1d–f). Upon ING1 knockdown, the proliferative abilities and invasiveness of SW480 cells were significantly increased, whereas the rate of apoptosis was significantly decreased compared with that in the control cells (Supplementary Fig. S1g–k). Conversely, ING1 overexpression in SW480 cells markedly reduced cell proliferation and invasion, while stimulating apoptosis (Supplementary Fig. S1g–k). These results indicated that ING1 may play an important tumour-suppressive role in colon cancer.

The inconsistent expression of ING1 at the protein and mRNA levels (Supplementary Fig. S1c) suggests that there is a posttranscriptional mechanism that regulates ING1 expression in colon cancer. MiRNAs are well-known posttranscriptional regulators. Therefore, we used TargetScan to identify putative miRNAs that could target ING1. Finally, we found 872 candidate miRNAs. Among them, we found ten highly expressed miRNAs in colon cancer tissue chip^5^ and identified five of them in YM500v3 (Supplementary Fig. S2a, b). Then, we collected data from TCGA to perform a meta-analysis to test whether the ten miRNAs were correlated with OS in deceased colon cancer patients. The results showed that higher miR-24-3p and miR-27b-3p were significantly related to shorter survival (Supplementary Fig. S2c). Then we verified the miR-24-3p and miR-27b-3p expression in 16 pairs of human colon cancer tissues and normal adjacent cancer tissues. The results showed that miR-24-3p was highly expressed in the cancer tissues and was inversely correlated with ING1 protein levels (*R* = −0.8326) (Supplementary Fig. S2d, e). However, miR-27b-3p was expressed at lower levels in the cancer tissues than normal tissues and had no correlation with ING1 protein levels (*R* = 0.1655) (Supplementary Fig. S2d, e). We used RNAhybrid to identify the minimum free energy hybridisation of the two binding sites between ING1 and miR-24-3p (−25.8 kcal/mol and −26.9 kcal/mol, respectively) (Fig. 1b).

Next, we performed luciferase assays to confirm whether miR-24-3p directly targets the 3′-UTR of ING1. Then, we co-transfected SW480 cells with recombinant firefly reporter plasmid, β-gal plasmid and either miR-24-3p mimic or inhibitor. The luciferase activity of SW480 cells was significantly inhibited by the miR-24-3p mimic, whereas the miR-24-3p inhibitor produced the opposite effect (Fig. 1c), suggesting that miR-24-3p can bind the 3′-UTR of ING1.

Subsequently, we measured the basal expression of miR-24-3p and ING1 in two colon cancer cell lines (SW480 and HT29), and found no differences between the two cell lines (Supplementary Fig. S3a–d). To investigate whether miR-24-3p can inhibit ING1 expression, we used miR-24-3p mimic and inhibitor to overexpress and knockdown miR-24-3p in SW480 and HT29 cells, respectively (Supplementary Fig. S3e). Regarding alterations in miR-24-3p levels, miR-24-3p overexpression dramatically decreased ING1 protein levels, whereas the miR-24-3p inhibitor increased the levels of ING1 protein (Fig. 1d, Supplementary Fig. S3f). However, changes in miR-24-3p levels had no considerable effect on ING1 mRNA levels (Supplementary Fig. S3g). To further validate the regulatory relationship between miR-24-3p and ING1, we transfected SW480 cells with miR-24-3p mimic, ING1 overexpression plasmid, or both. ING1 expression was significantly increased upon co-transfection of ING1 overexpression plasmid and miR-24-3p mimic compared with that upon transfection of miR-24-3p mimic alone (Fig. 1e, Supplementary Fig. S3h), indicating that ING1 overexpression fully recapitulated the inhibitory effect of miR-24-3p on ING1. Taken together, these results suggest that miR-24-3p suppresses ING1 protein expression by binding the 3′-UTR of ING1.

To determine the biological effects of miR-24-3p leading to the downregulation of ING1 in colon cancer, we examined the changes in cell proliferation, apoptosis and invasion by transfecting miR-24-3p mimic or inhibitor into SW480 and HT29 cells. The miR-24-3p mimic significantly accelerated the proliferative ability of SW480 and HT29 cells, whereas the miR-24-3p inhibitor impeded this ability (Supplementary Fig. S4a, b). Consistent with these findings, compared with the control, miR-24-3p mimics decreased SW480 and HT29 cell apoptosis, whereas miR-24-3p inhibitor increased apoptosis (Supplementary Fig. S4c, d). In addition, miR-24-3p overexpression promoted SW480 and HT29 cell invasion, whereas miR-24-3p knockdown decreased SW480 and HT29 cell invasion (Supplementary Fig. S5a–c).

To confirm whether the effects of miR-24-3p on SW480 proliferation, invasion and apoptosis were due to direct targeting of ING1, we performed cell recovery experiments to restore miR-24-3p-inhibited ING1 expression by transfecting an ING1 overexpression plasmid into the cells. Ectopic expression of ING1 completely eliminated the promoting effects of miR-24-3p on the proliferation and invasion of SW480 cells, resulting in a higher percentage of apoptotic cells than that in SW480 cells with miR-24-3p overexpression (Fig. 1f–i). These results suggested that miR-24-3p functions as an oncomiR to promote SW480 proliferation and invasion, and suppress apoptosis by targeting ING1 in colon cancer cells.

To evaluate the effect of miR-24-3p-mediated inhibition of ING1 on colon cancer growth in vivo, we prepared a colon cancer orthotopic xenograft mouse model by injecting SW480 cells overexpressing miR-24-3p and/or ING1 into the axilla of nude mouse. After 4 weeks, there were no significant differences in the body weights of the mouse, miR-24-3p overexpression increased tumour weights, while ING1 overexpression significantly decreased tumour weights at this time point (Fig. 1j, Supplementary Fig. S6a). Ectopic ING1 expression attenuated the enhancement of tumour growth induced by miR-24-3p (Fig. 1j, Supplementary Fig. S6a). Subsequently, to determine the levels of miR-24-3p and ING1 in the xenografts, we extracted total RNA and proteins from the tumour. As expected, the miR-24-3p and ING1 protein levels in the miR-24-3p-overexpressing and ING1-overexpressing groups, respectively, were higher than those in the control group (Supplementary Fig. S6b–d). The ING1 plasmid effectively restored the ING1 protein level suppressed by miR-24-3p (Supplementary Fig. S6c, d). Haematoxylin and eosin (H&E) staining of the xenograft tumours showed that, compared with the control group, the miR-24-3p-overexpressing group had more cell mitosis and stronger invasiveness, while the ING1-overexpressing group had less mitosis and a larger area of cell necrosis; however, xenografts overexpressing both miR-24-3p and ING1 showed lower cell mitosis and weaker invasiveness than xenografts overexpressing miR-24-3p alone (Supplementary Fig. S6e). Immunohistochemical staining for ING1, Ki-67, caspase3 and vimentin showed that tumours overexpressing miR-24-3p had less expression of ING1 and caspase3 but higher expression of Ki-67 and vimentin than did tumours from the control group (Supplementary Fig. S6e–i). ING1 overexpression restored the effects of miR-24-3p, resulting in decreased tumour growth and invasion and increased tumour apoptosis (Supplementary Fig. S6e–i). Consistent with the in vitro studies, the in vivo studies revealed that miR-24-3p exerted its oncogenic effect on colon carcinogenesis by targeting ING1. Phosphorylation of ING1 regulates ING1’s subcellular localisation, which is important for its biological function. Our results showed that miR-24-3p decreased the expression of ING1 in both the locus and the cytoplasm (Supplementary Fig. S6e). This indicates that phosphorylation/dephosphorylation may not be a regulatory pathway in this axis.

Taken together, these results suggest that miR-24-3p posttranslationally regulates ING1 expression to promote colon carcinogenesis. These findings elucidate the molecular mechanisms involved in ING1 downregulation and provide important insight into the controversial roles of miR-24-3p in colon cancer. Therefore, our data highlight the potential for targeting miR-24-3p as a therapeutic strategy to enhance the treatment of colon cancer.

## Supplementary information

Supplementary_Materials
