# Supplementary material for: miR-24-3p promotes colon cancer progression by targeting ING1
Source: Signal Transduct Target Ther. 2020 Aug 25;5:171. doi: 10.1038/s41392-020-0206-y (PMC7447644; doi:10.1038/s41392-020-0206-y)
Supplement: Supplementary file 1 — Supplementary_Materials [file 41392_2020_206_MOESM1_ESM.docx]

Supplementary Materials for

miR-24-3p promotes colon cancer progression by targeting ING1

Zhiying Gao^1,2†^, Likun Zhou^1†^, Shiyu Hua^1^, Huan Wu^3^, Laizhi Luo^4^, Libo Li^2^, Shuping Wang^5^, Yanqing Liu^1^, Zhen Zhou^1*^ and Xi Chen^1*^

1. State Key Laboratory of Pharmaceutical Biotechnology, Collaborative Innovation Center of Chemistry for Life Sciences, Jiangsu Engineering Research Center for MicroRNA Biology and Biotechnology, NJU Advanced Institute for Life Sciences (NAILS), School of Life Sciences, Nanjing University, Nanjing, Jiangsu 210046, China;
2. Research Institute of Medicine and Pharmacy, Qiqihar Medical University, Qiqihar, Heilongjiang, 161006, China;
3. The Department of Ultrasound of Sun Yat-Sen Memorial Hospital, Sun Yat-Sen University, Guangzhou, Guangdong, 510000, China;
4. The Sixth Clinical Medical College of Capital Medical University, Beijing, 100029, China;
5. The First Hospital of Qiqihar, Qiqihar Medical University, Qiqihar, Heilongjiang, 161006, China.

^†^These authors contributed equally to this work.

Correspondence to: Xi Chen (xichen@nju.edu.cn) and Zhen Zhou (zhenzhou@nju.edu.cn)

**This PDF file includes:**

Materials and Methods

Supplementary Text

Figures. S1 to S6

Materials and Methods

Human tissue samples

Human colon cancer tissues and paired adjacent noncancerous colon tissues were obtained from Nanjing University Medical School (Nanjing, China). Written consent was provided by all the patients (or their guardians), and the Ethics Committee of Nanjing University approved all aspects of this study. Tissue samples excised during surgery were immediately stored in liquid nitrogen at -80°C. The clinical features of the patients are listed in supplementary Table S1.

Cell culture

HT29 and SW480 cells were purchased from the Shanghai Institute of Cell Biology of the Chinese Academy of Sciences (Shanghai, China) and cultured in RPMI 1640 medium (Gibco, Carlsbad, CA, USA) supplemented with 10% fetal bovine serum (Gibco) in a humidified incubator at 37°C in 5% CO_2_.

RNA isolation and quantitative real-time polymerase chain reaction (qRT-PCR)

Total RNA was extracted from the cultured cells and tissues using TRIzol reagent (Invitrogen, CA, USA). miR-24-3p levels were quantified on an Applied Biosystems 7500 Sequence Detection System using TaqMan miRNA Assay Probes (Applied Biosystems, Foster City, CA). U6 snRNA was used as an internal control. The relative expression of miR-24-3p normalized to U6 expression was calculated with the equation 2^-ΔΔCT^, in which ΔΔCT = (CT miR-24-3p − CT U6) tumor− (CT miR-24-3p− CT U6) control.

To quantify ING1 and GAPDH mRNA expression, oligo (dT)18 primers (TaKaRa) were used to reverse transcribe total RNA into cDNA. Then, qRT-PCR was performed using SYBR Green (Invitrogen) and specific primers for ING1 and GAPDH. The primer sequences were as follows: ING1, (sense) 5’- AACAACGAGAACCGTGAGAAC-3’ and (antisense) 5’-TGGTTGCACAGACAGTACGTG-3’; and GAPDH, (sense) 5’-ACCACAGTCCATGCCATCAC-3’ and (antisense) 5’-TCCACCACCCTGTTGCTGTA-3’.

miRNA target prediction

The TargetScan algorithm (http://www.targetscan.org/vert_72/) was used to search potential miRNAs that could target ING1. RNAhybrid (https://omictools.com/rnahybrid-tool) was used to identify the minimum free energy hybridization of RNA.

Meta-analysis of miRNA expression in colon cancer

We used the YM500v3 online database (http://120.110.158.132:8787/ym500v3/) and colon cancer chip^5^ to analyze miRNAs expression in colon cancer tissues (primary or recurrent solid tumors), adjacent noncancerous colon tissues and normal solid tissues. YM500v3 database compared the miR-24-3p expression profiles between 8 normal solid colon tissues and 398 primary solid colon tumors. Data pertaining to the OS of colon cancer patients stratified by miRNAs expression level was downloaded from the TCGA data portal (https://tcga-data.nci.nih.gov/docs/publications/lusc_2012/). We split patients into high or low expression based on the mean expression of miRNAs in deceased patients.

Western blot analysis (WB)

RIPA lysis buffer (Beyotime, Shanghai, China) freshly mixed with a protease and phosphatase inhibitor cocktail (Thermo Scientific, Rockford, Cambridge, MA) was used to isolate proteins from cells or tissues. Proteins were separated by SDS-PAGE through 10% gels (Bio-Rad). Antibodies for Western blotting were as follows: anti-ING1b purchased from Cell Signaling Technology (14625; CST, USA), and anti-GAPDH purchased from Santa Cruz Biotechnology (sc-365062; Santa Cruz, CA, USA).

Overexpression or knockdown of miR-24-3p

Synthetic miR-24-3p mimic and inhibitor and corresponding negative control RNAs were purchased from RiboBio (Guangzhou, China). Colon cancer cells were transfected with RNA oligoribonucleotides using Lipofectamine 2000 (Invitrogen, CA, USA). Generally, 100 pmol of miR-24-3p mimic, inhibitor or scrambled negative control RNAs was used for each transfection. After 48 h, the transfected cells were harvested for subsequent experiments.

Plasmid construction and siRNA interference assay

A vector encoding the open reading frame of human ING1 without the 3’-UTR (EX-NEG-M98) was obtained from GeneCopoeia (Germantown, MD, USA). An empty plasmid served as a negative control (control plasmid). siRNAs designed to specifically silence ING1 were purchased from RiboBio (Guangzhou, China) with a scrambled siRNA serving as a control. The siRNA sequence for ING1 was GCGACGAGAAGATCCAGAT. The overexpression plasmids and siRNAs were transfected into colon cancer cells using Lipofectamine 2000 (Invitrogen). Total RNA and protein were isolated 48 h after transfection and were assessed by quantitative RT-PCR and Western blot, respectively.

Luciferase reporter assay

The ING1-3’-UTR-WT vector was constructed by inserting the amplified 3’-UTR of human ING1 into a luciferase reporter vector. The sites that interact with the miR-24-3p seed sequence were mutated from TGAGCCA to ACTCGGT to create a mutant ING1 3’-UTR, which was inserted into a luciferase reporter plasmid to construct the ING1-3’-UTR-MUT vector. Luciferase reporter plasmid (1 μg), β-galactosidase (β-gal) expression plasmid (1 μg), and 100 pmol of the miR-24-3p mimic, inhibitor or scrambled negative control RNA were cotransfected into colon cancer cells using Lipofectamine 2000, with the β-gal plasmid serving as a transfection control. After 24 h, the cells were analyzed for luciferase activity on a Modulus Luminometer (Turner Biosystems, Sunnyvale, USA) using luciferase assay kits (Promega, Madison, WI, USA).

Cell proliferation assay

For the CCK-8 assay, SW480 cells were seeded at 2 × 10^4^ cells per well in 96-well plates and incubated overnight in RPMI 1640 supplemented with 10% FBS. The cell proliferation index was measured using a Cell Counting Kit-8 assay (CK04-500, Dojindo, Japan) at 12, 24, 36, 48, and 60 h posttransfection according to the manufacturer’s instructions. Absorbance was measured at a wavelength of 450 nm.

Apoptosis assay

The apoptosis of SW480 cells was determined using the FITC-Annexin V Apoptosis Detection Kit I (BD Biosciences) based on the procedures provided by the manufacturer. Transfected SW480 cells were cultured in serum-free DMEM for 24 h. After the cells were harvested, they were washed with cold PBS and resuspended in 1 × binding buffer, followed by an incubation with FITC-Annexin V and propidium iodide (PI) in the dark for 15 min. The apoptotic cells were calculated with fluorescence-activated cell-sorting (FACS) on a flow cytometer (BD Biosciences, San Jose, CA, USA). The individual populations can be defined using quadrant gates, which automatically quantify the number of cells in each quadrant as follows: quadrant 4 = non-apoptotic cells (Annexin V-FITC negative/PI negative), quadrant 3 = early apoptotic cells (Annexin V-FITC positive/PI negative), quadrant 2 = late apoptotic/necrotic cells (Annexin V-FITC positive/PI positive) and quadrant 1 =dead cells (Annexin V-FITC negative/PI positive). The percentage of apoptotic cells is determined by adding the percentages of cells in each of the Q2 and Q3 quadrants.

Transwell invasion assay

Cell invasion assays were performed using Millipore 24-well Millicell (Millipore) plates containing a Transwell membrane with an 8-μm pore diameter. The membrane side exposed to the lower chamber was coated with 10 μg/mL fibronectin (Gibco). Cells were harvested 24 h after transfection and suspended in serum-free RPMI 1640 culture medium. The cells were then added to the upper chamber (4 × 10^4^ cells/well), and 0.5 mL RPMI 1640 containing 20% FBS was added to the lower chamber. The Transwell-containing plates were incubated for 24 h in the incubator, after which the cells that had migrated to the lower surface of the membrane were fixed with 4% paraformaldehyde for 25 min at room temperature. The membrane was washed 3 times with PBS and stained with 0.1% crystal violet in methanol for 15 min at room temperature. Cells remaining on the upper surface of the membrane were gently removed with a cotton swab. The lower surfaces were viewed under a fluorescence microscope (BX51 Olympus, Japan), and the number of cells in five random fields per chamber was counted.

Establishment of tumor xenografts in mouse

Athymic BALB/c male nude mouse (4 weeks old) were purchased from the Model Animal Research Center of Nanjing University (Nanjing, China) and randomly divided into 3 groups (3 mouse per group). SW480 cells either were infected with a control lentivirus or a lentivirus overexpressing miR-24-3p or were transfected with an ING1 overexpressing plasmid. After plasmid transduction, the SW480 cells were uniformly suspended in PBS at a final concentration of 1× 10^7^ per mL. For each mouse, 0.1 mL of cell suspension solution (1× 10^6^ cells) was subcutaneously injected according to standard protocols. The needle was inserted into the axilla of the right hind leg at a 45-degree angle and a 5 mm depth, midway down. After the mouse were sacrificed and photographed 28 days postinjection, the xenograft tumors were removed and weighed. Part of the tissue was used for extracting total RNA and protein, and the remainder of the tissue was made into tumor section slides for H&E and IHC staining for ING1, Ki-67, caspase 3 and vimentin. All animal care and handling procedures were performed in accordance with the National Institutes of Health Guide for the Care and Use of Laboratory Animals.

Statistical analysis

All of the images of the Western blot assays, Transwell invasion assay or animal experiments were representative of at least three independent experiments or staining results. The quantitative RT-PCR, luciferase reporter, and proliferation assays were performed in triplicate, and each individual experiment was repeated several times. The results are presented as mean ± s.e.m of at least three independent experiments. Observed differences were considered statistically significant at *p* < 0.05 based on Student’s t-test.

Supplementary Text

Abbreviations

ING1, Inhibitor of Growth 1;

miRNA, microRNA;

3’-UTR, 3’-untranslated region;

TCGA, The Cancer Genome Atlas;

OS, overall survival;

H&E staining, Haematoxylin and eosin staining;

IHC staining, Immunohistochemical staining.

Acknowledgements

This work was supported by grants from Heilongjiang Province Department of Education (UNPYSCT-2018035).

Figure. S1.

**
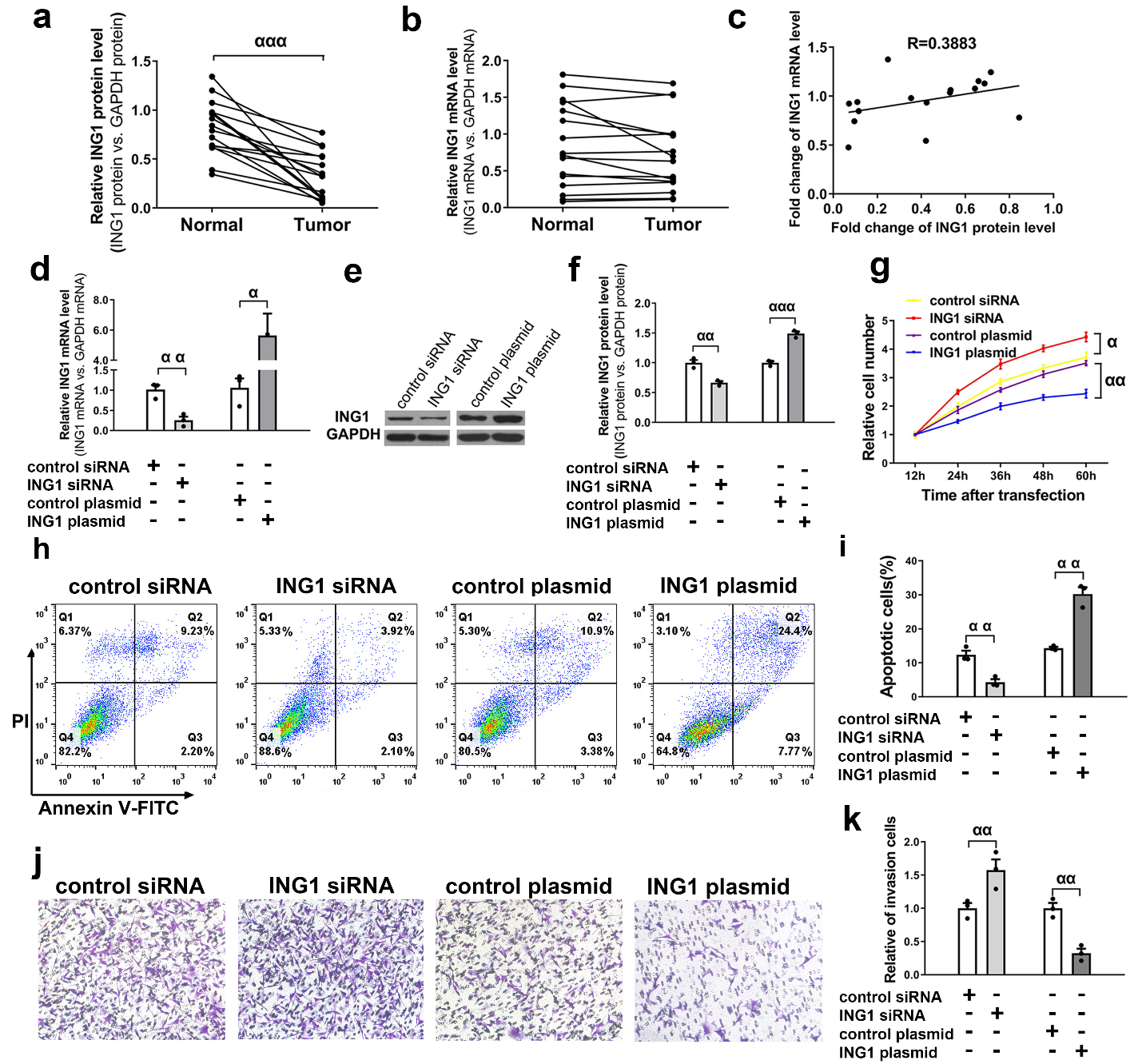
**

**Fig.S1 Expression levels of ING1 in colon cancer tissues and its role in colon cancer cells.** **(a and b)** WB and qRT-PCR analysis: ING1 protein and mRNA levels in 16 paired colon cancer (T) and normal adjacent tissue (N) samples. **(c)** Pearson’s correlation scatter plot of the fold changes of ING1 protein and mRNA levels in colon cancer tissue pairs. **(d-f)** qRT-PCR and WB analysis: The efficient knockdown and overexpression of ING1 in the SW480 cells transfected with ING1 siRNA or an ING1 overexpression plasmid. **(g-k)** Effects of ING1 on the proliferation, apoptosis and invasion of SW480 cells transfected with control siRNA, ING1 siRNA, control plasmid or an ING1 overexpression plasmid. **g:** CCK8 assay; **h:** Apoptosis assay; **i:** Quantitative analysis of apoptosis assays. **j:** Transwell invasion assay; **k:** Quantitative analysis of transwell invasion assays. Data are showen as mean ± s.e.m. in **d, f, g, i and k** with data points from independent experiment in **d, f, i and k**. α *p* < 0.05; αα *p* < 0.01; ααα *p* < 0.001

Figure. S2.

**
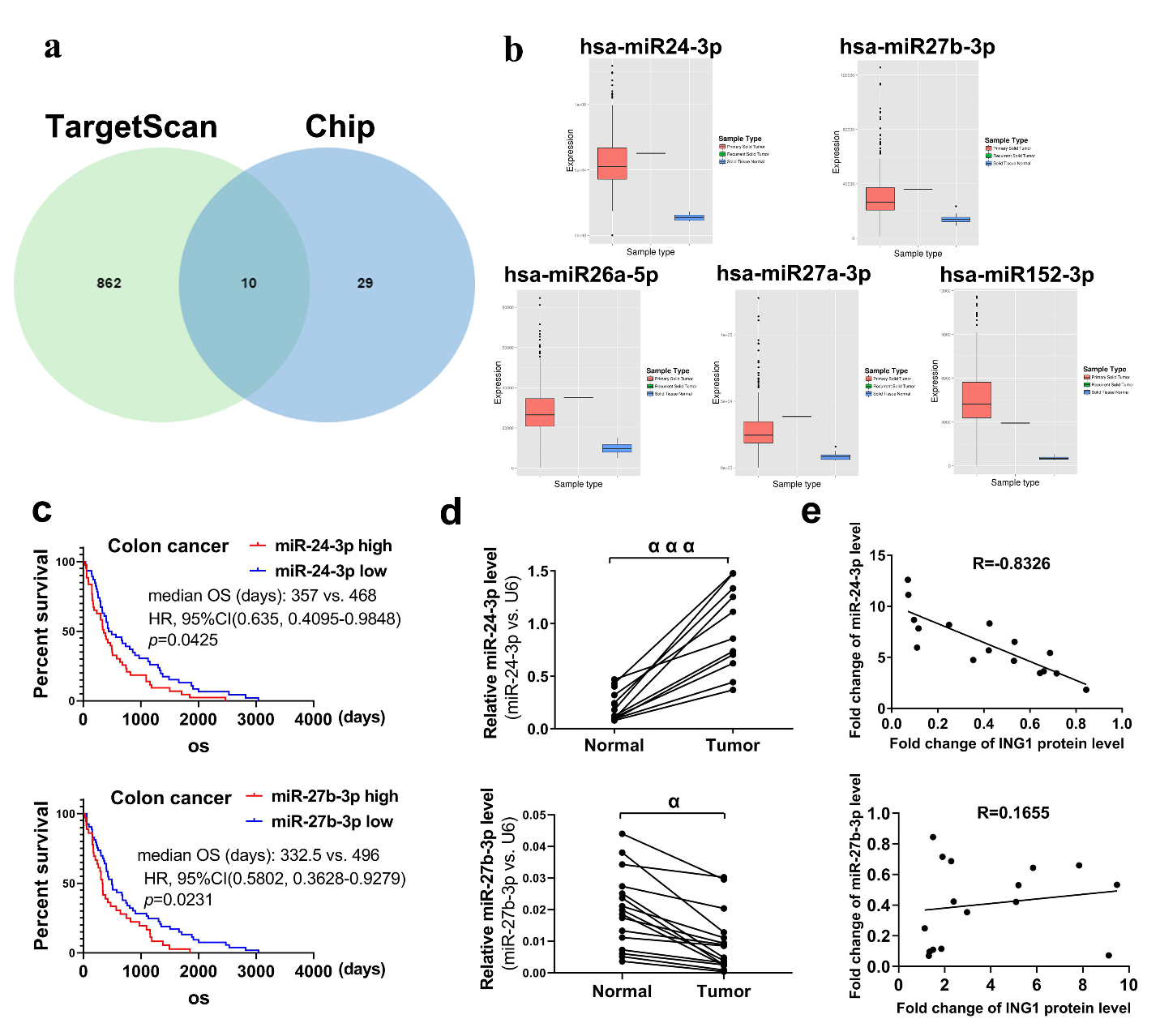
**

**Fig.S2 ING1 is a potential target gene of miR-24-3p.** **(a)** A Venn diagram was used to search for potential miRNAs that could target ING1 in colon adenocarcinoma. **(b)** Levels of candidate miRNAs in colon adenocarcinoma tissue pairs from YM500v3. Red color: Primary solid tumor; green color: Recurrent solid tumor; blue color: Solid tissue Normal. **(c)** Influence of miR-24-3p and miR-27b-3p expression on overall survival in colon adenocarcinoma patients from TCGA dataset. **(d)** qRT-PCR analysis of miR-24-3p and miR-27b levels in 16 paired colon cancer and normal adjacent tissue samples. **(e)** Pearson’s correlation scatter plot between miR-24-3p/miR-27b-3p and ING1 protein levels in colon cancer tissue pairs. α *p* < 0.05; ααα *p* < 0.001

Figure. S3.

**
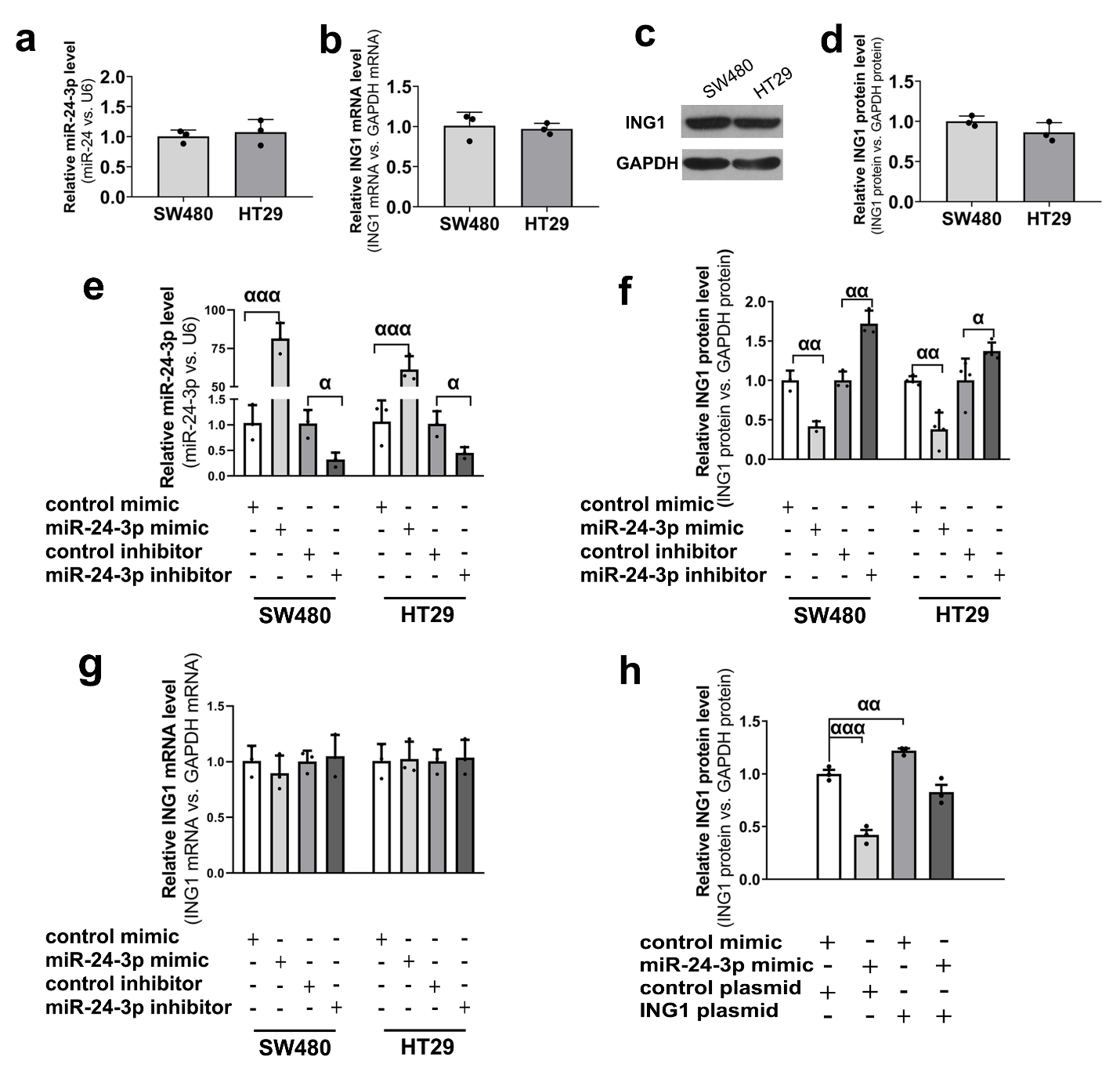
**

**Fig.S3 miR-24-3p can inhibit** **ING1 expression.** **(a and b)** qRT-PCR analysis: Basic levels of miR-24-3p and ING1 mRNA in SW480 and HT-29 cells. miR-24-3p and ING1 mRNA levels of SW480 was set as control. **(c and d)** WB and quantitative analysis: Basic levels of ING1 protein in SW480 and HT-29 cells. ING1 protein level of SW480 was set as control. **(e)** qRT-PCR analysis of miR-24-3p levels in SW480 and HT29 cells after treatment with miR-24-3p mimic or inhibitor. **(f-g)** WB and qRT-PCR analysis of ING1 levels in SW480 and HT29 cells after treatment with miR-24-3p mimic or inhibitor. **(h)** Quantitative analysis of WB: ING1 plasmid could effectively restored the ING1 protein level suppressed by miR-24-3p. Data are showen as mean ± s.e.m. with data points from independent experiment in **a-b and d-h.** α *p* < 0.05; αα *p* < 0.01; ααα *p* < 0.001

Figure. S4.

**
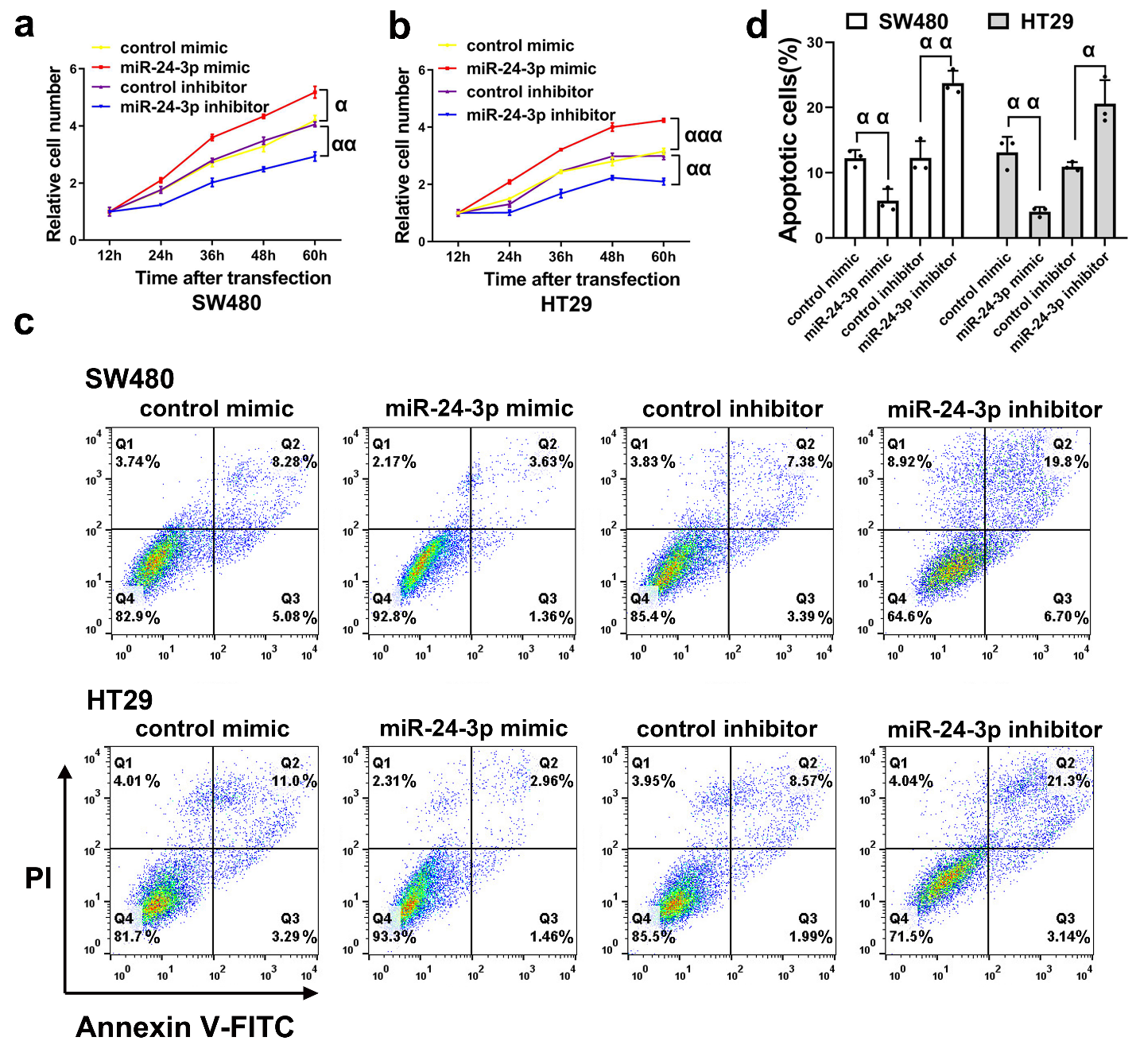
**

**Fig.S4 miR-24-3p promotes colon cancer cell proliferation and inhibits colon cancer cell apoptosis. (a and b)** CCK8 assay: miR-24-3p promotes SW480 and HT29 proliferation. **(c and d)** Apoptosis assay and statistical analysis: miR-24-3p inhibits SW480 and HT29 apoptosis. Data are showen as mean ± s.e.m. in **a-b and d** with data points from independent experiment in **d.** α *p* < 0.05; αα *p* < 0.01; ααα *p* < 0.001

Figure. S5.

**
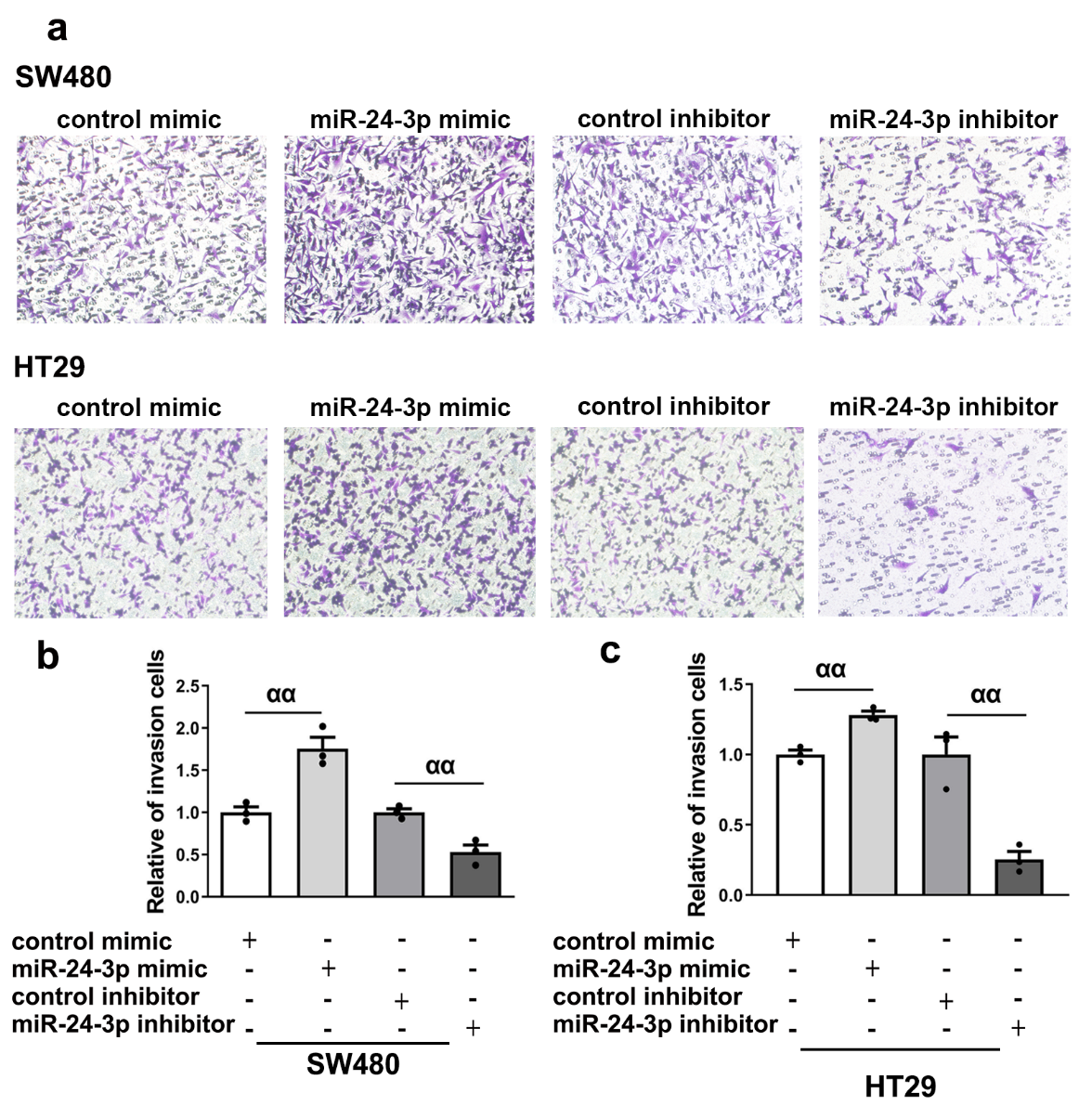
**

**Fig.S5 miR-24-3p promotes colon cancer cell invasion. (a-c)** Tanswell invasion assays were performed in SW480 and HT29 cells that were transfected with equal doses of control mimic, miR-24-3p mimic, control inhibitor or miR-24-3p inhibitor. **a:** Representative images of transwell assays; **b and c**: Quantitative analysis of transwell invasion assays. Data are showen as mean ± s.e.m. with data points from independent experiment in **b-c**. αα *p* < 0.01

Figure. S6.

**
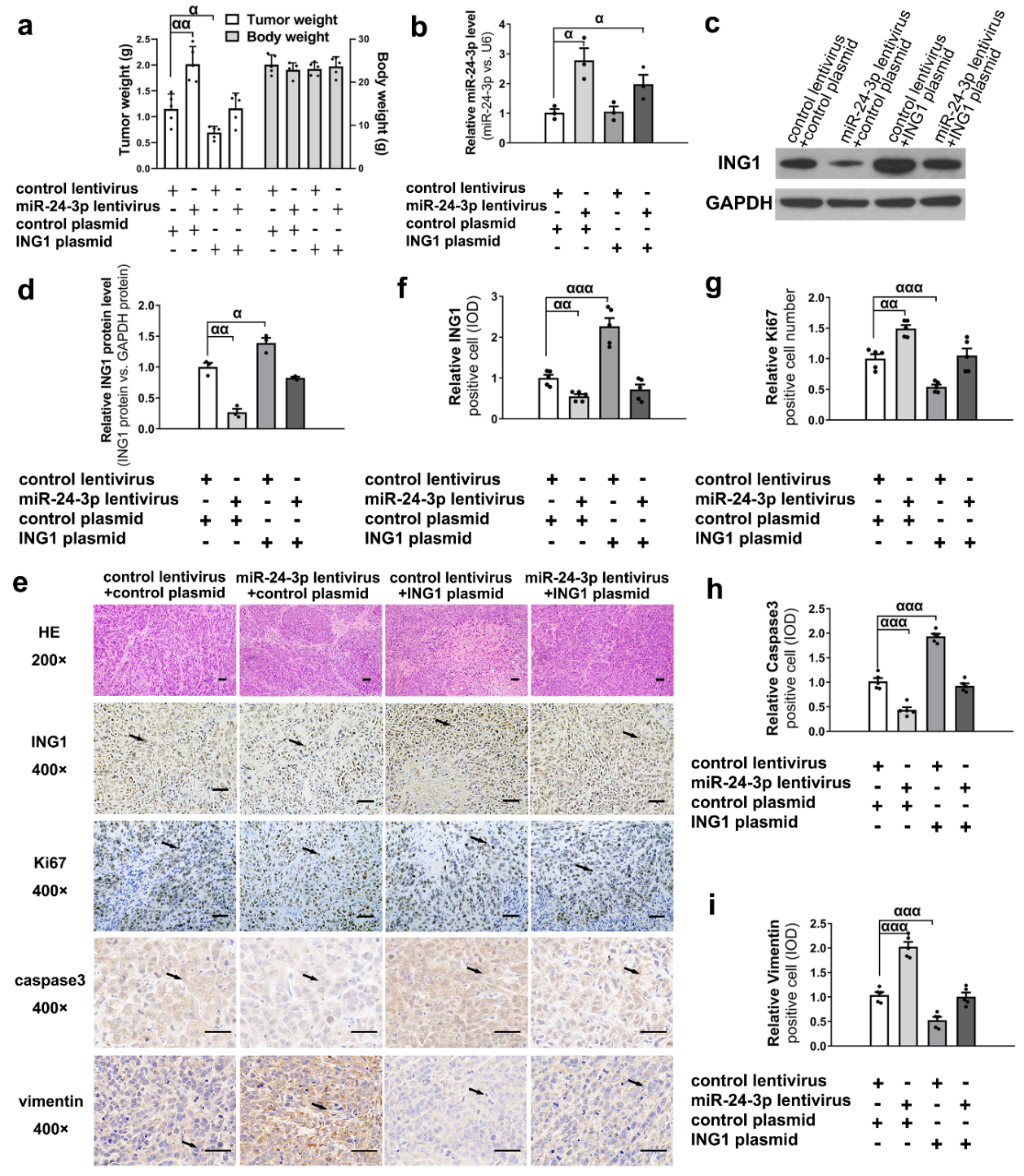
**

**Fig.S6 miR-24-3p promotes colon cancer growth and invasion and reduces apoptosis protein in vivo by targeting ING1. (a)** Quantitative analysis of xenograft tumour weights and body weights of mouse. **(b)** qRT-PCR analysis of miR-24-3p levels in colon cancer xenografted tumours. **(c-d)** WB and statistical analysis: ING1 protein levels in colon cancer xenografted tumours. **(e)** Representative image of H&E-stained sections and IHC staining for ING1, Ki67, caspase3 and vimentin in tumors from the implanted mouse (arrows indicate positively-stained cells). Scale bar= 50 μm. **(f-i)** Quantitative analysis of IHC staining for ING1, Ki67, caspase3 and vimentin in tumors from the implanted mouse. Data are showen as mean ± s.e.m. with data points from individual mouse in **a-b, d and f-i.** α *p* < 0.05; αα *p* < 0.01

Table S1.

Clinical features of colon cancer patients.

| Case number | Age | Gender | TNM stage | Cancer subtype |
| --- | --- | --- | --- | --- |
| 1 | 53 | F | III | colon adenocarcinoma |
| 2 | 58 | M | III | colon adenocarcinoma |
| 3 | 61 | M | III | colon adenocarcinoma |
| 4 | 59 | F | III | colon adenocarcinoma |
| 5 | 67 | F | III | colon adenocarcinoma |
| 6 | 49 | M | III | colon adenocarcinoma |
| 7 | 54 | F | IV | colon adenocarcinoma |
| 8 | 50 | M | IV | colon adenocarcinoma |
| 9 | 66 | F | III | colon adenocarcinoma |
| 10 | 68 | M | IV | colon adenocarcinoma |
| 11 | 53 | F | III | colon adenocarcinoma |
| 12 | 50 | M | IV | colon adenocarcinoma |
| 13 | 71 | M | III | colon adenocarcinoma |
| 14 | 65 | F | III | colon adenocarcinoma |
| 15 | 60 | F | III | colon adenocarcinoma |
| 16 | 47 | M | III | colon adenocarcinoma |
